# Electrical stimulation of macaque lateral prefrontal cortex modulates oculomotor behavior indicative of a disruption of top-down attention

**DOI:** 10.1038/s41598-017-18153-9

**Published:** 2017-12-18

**Authors:** Philipp Schwedhelm, Daniel Baldauf, Stefan Treue

**Affiliations:** 1 Cognitive Neuroscience Laboratory, German Primate Center Kellnerweg 4, 37077 Goettingen, Germany; 2 Center for Mind and Brain Sciences, University of Trento Via delle Regole 101, 38123 Mattarello, TN Italy; 3 Bernstein Center for Computational Neuroscience, Am Fassberg 17, 37077 Goettingen, Germany; 4 McGovern Institute for Brain Research, Massachusetts Institute of Technology MIT Bldg 46-3160, 43 Vassar Street, Cambridge, MA 02139 USA; 5 Faculty of Biology and Psychology, University of Goettingen Wilhelm-Weber-Str. 2, 37073 Goettingen, Germany; 6 Leibniz ScienceCampus Primate Cognition, German Primate Center Kellnerweg 4, 37077 Goettingen, Germany; 7 Present Address: Center for Mind and Brain Sciences, University of Trento Via delle Regole 101, 38123 Mattarello, TN Italy

## Abstract

The lateral prefrontal cortex (lPFC) of primates is hypothesized to be heavily involved in decision-making and selective visual attention. Recent neurophysiological evidence suggests that information necessary for an orchestration of those high-level cognitive factors are indeed represented in the lPFC. However, we know little about the specific contribution of sub-networks within lPFC to the deployment of top-down influences that can be measured in extrastriate visual cortex. Here, we systematically applied electrical stimulations to areas 8Av and 45 of two macaque monkeys performing a concurrent goal-directed saccade task. Despite using currents well above saccadic thresholds of the directly adjacent Frontal Eye Fields (FEF), saccades were only rarely evoked by the stimulation. Instead, two types of behavioral effects were observed: Stimulations of caudal sites in 8Av (close to FEF) shortened or prolonged saccadic reaction times, depending on the task-instructed saccade, while rostral stimulations of 8Av/45 seem to affect the relative attentional weighting of saccade targets as well as saccadic reaction times. These results illuminate important differences in the causal involvement of different sub-networks within the lPFC and are most compatible with a stimulation-induced biasing of stimulus processing that accelerates the detection of saccade targets presented ipsilateral to stimulation through a disruption of contralaterally deployed top-down attention.

## Introduction

The lateral prefrontal cortex (lPFC) of primates plays a pivotal role in representing task goals and behavioral priorities. This includes the representation of rules and categories^1–5^, the memory of to-be-searched-for objects or features^6–8^, as well as spatial representations of behavioral relevant objects^9^ and locations^10^. All of these are crucial for successful interactions with the environment, e.g. in visual search or the coordination of goal-directed movements, such as saccades. In natural, free-viewing search tasks, for example, it has been shown that the selection of relevant features, objects, and locations occurs first in lPFC and is only later communicated to visual areas^11–14^.

**Figure 1:**
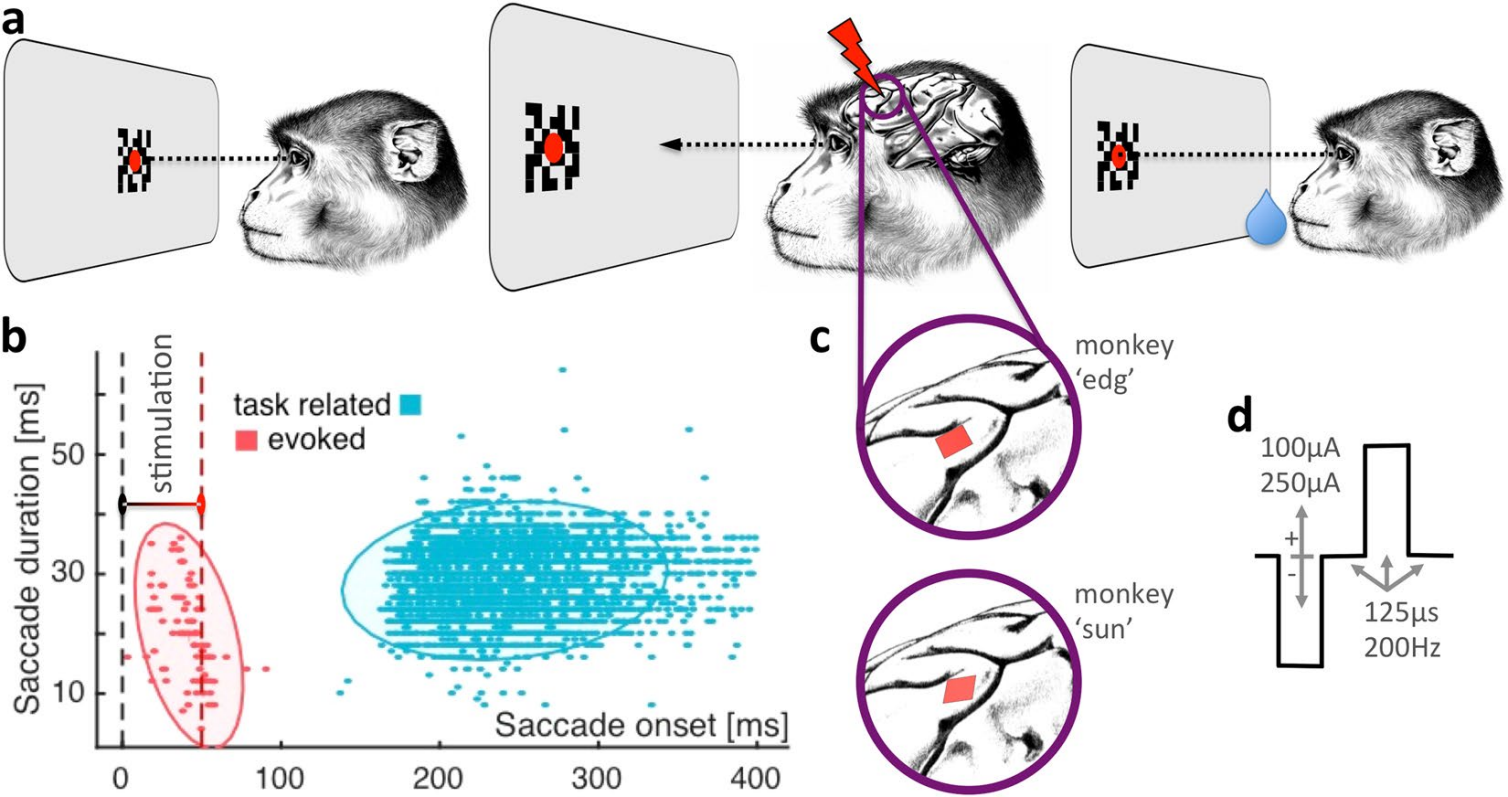
Experimental paradigm. Monkeys performed a visually guided center-out saccade task while the lateral prefrontal cortex was electrically stimulated. **(a)** Each trial was initiated when the animals foveated a central fixation point. After 800 ms, the fixation point jumped left- or rightwards along the horizontal meridian (±7 deg), and the monkeys had to make a saccade to the target location to receive a liquid reward. **(b)** Saccades observed fell into two categories: saccades initiated >100 ms relative to fixation point jump were considered task related (cyan) and saccades initiated between 0–100 ms were classified as directly evoked by the stimulation (red). Ellipses indicate 95% confidence boundaries for saccade parameters. **(c)** Electrical stimulation was delivered through 96-channel Utah arrays (red shaded areas), implanted in each monkeys left area 8Av. **(d)** We stimulated single electrodes blockwise on 50% of all trials. In stimulation trials, 10 biphasic pulses with a frequency of 200 Hz were delivered, with a current of either 100 or 250 μA. (**a/c**) Drawings by: Klaus Lamberty, Deutsches Primatenzentrum GmbH.

The lPFC, which in non-human primates is delineated by the junction of the two major frontal sulci, the arcuate and the principal (Fig. 1c), contains cortical patches carrying both spatial and non-spatial sensory information. Since those representations often lie side by side, the complex cascade of neural events within lPFC has recently received increased attention and was linked to high-level cortical functions during the preparation and execution of a wide variety of tasks^15–17^.

In particular, the frontal eye fields (FEF) have a strong spatiotopic organization and are a core node of the saccade generation network^18–20^. Electrical stimulation of the FEF typically evokes saccades, while sub-threshold microstimulation causes more subtle behavioral effects^21,22^ and changes in the responses of visual cortical neurons that resemble the modulatory effects of spatial attention^23–26^.

Directly anterior to the FEF of the macaque monkey lies the rostral part of area 8 A^27,28^ which can be further subdivided into a dorsal and ventral part^29–31^. Area 8Av is heavily interconnected with both visual cortical areas and other prefrontal areas^32–35^, pointing at its central role in visually-guided tasks.

Recent electrophysiological investigations of neurons along the border between areas 8Av and 45 (here termed 8Av/45) indicate this functional region’s involvement in encoding task-relevant visual information^36–38^ as well as behaviorally relevant information related to task rules^1,39^. It is thus hypothesized to be involved in the deployment of visual attention^1,40,41^.

In fact, the functionally defined ventral prearcuate region (VPA)^42^ has recently been suggested as a likely source of feature-based attention, which is known to modulate responses across most of visual cortex^13,43–48^. VPA partially overlaps with the rostral portion of 8Av/45, which thus lies directly in between two cortical patches (FEF and VPA) that are hypothesized to be responsible for the deployment of spatial and feature-based attention, respectively.

It has long been known that spatial attention facilitates the detection of stimuli at attended locations and thus lowers reaction times for those stimuli^49^. Here, we attempt to characterize the *causal* role of macaque 8Av/45 in such sensory facilitation, as well as the role of putative neural sub-networks within this region of the lPFC. In this matter, instantaneously evoked motor responses need to be separated from behavioral effects that would be caused by disruptions of the attentional system. We address this by systematically applying electrical stimulation through grids of electrodes spanning across 8Av/45 of two macaque monkeys, while the animals were engaged in a left-right, center-out visually guided saccade task.

## Results

### Stimulation evoked saccades only on caudal-most sites

We electrically stimulated each available electrode of two 96-channel electrode arrays, which were implanted in the left lateral prefrontal cortex of two macaque monkeys, at a location just ventral to the caudal end of the principal sulcus (here termed 8Av/45). Stimulation was delivered while the animals were performing a center-out (left/right) visually guided saccade task (Fig. 1). Each saccade (determined by a velocity threshold of 100 deg/s) initiated within 100 ms from stimulation onset and lasting no longer than another 75 ms was considered evoked by the stimulation (Fig. 1b–d). We included an electrode site in the analysis when saccades were evoked in at least 3 out of 10 stimulation trials (10 biphasic pulses, 200 Hz).

**Figure 2:**
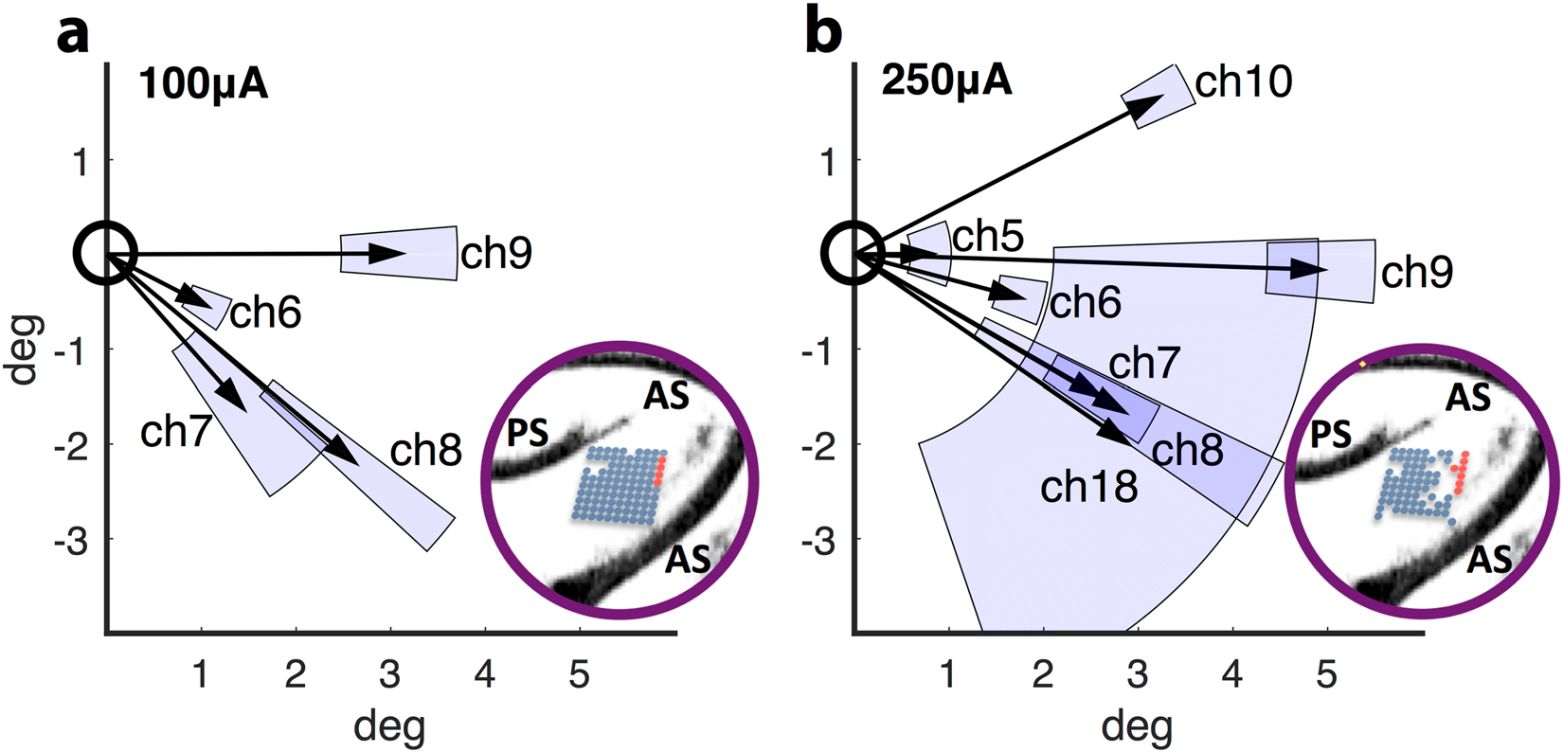
Saccades directly evoked by stimulation. In one animal, we found a group of spatially clustered electrodes whose stimulation directly evoked saccades. During stimulation with a 100 μA current (**a**) saccades were robustly evoked on 4 channels. When a higher current of 250 μA was used, the stimulation of adjacent electrodes also evoked saccades **(b**). The relevant electrodes were located on the caudal border of the array, closest to the concavity of the arcuate sulcus (see inserts, red electrodes). The amplitudes of directly evoked saccades significantly increased with stimulation current. Arrows in both plots represent the average evoked saccade vector and blue shaded areas represent the respective confidence intervals for saccadic end-points. (**a/b**) Drawings by: Klaus Lamberty, Deutsches Primatenzentrum GmbH.

Such evoked saccades were only observed in one animal and only in a small spatial cluster of electrodes. The saccades were exclusively directed towards the contralateral hemifield, relative to the stimulated hemisphere (see Fig. 2).

Notably, a high stimulation current (250 μA, Fig. 2b) evoked saccades on more electrodes than a lower current (100 μA, Fig. 2a), but the additional stimulation sites with evoked saccades were located no more than 400 μm away from sites with evoked saccades during low current stimulation. However, due to connectivity issues with some electrodes during stimulation with 250 μA (see Fig. 2 small inserts) and the fact that electrodes with evoked saccades were located at the border of the array, we were not able to exactly estimate the spatial extent of the saccade-evoking patch of cortex. Still, the frequency of saccades evoked by stimulation of the four electrodes with evoked saccades during stimulation with both currents significantly increased as a function of current (p = 0.003, odds ratio = 0.175; Fishers exact test).

To determine the effects of stimulation current on saccade direction, we restricted our analysis to electrodes with evoked saccades in both stimulation conditions and calculated a 2-way ANOVA for circular data^50^ with electrode location and stimulation current as explanatory variables. We found a highly significant effect of electrode site on saccade direction (p ≪ 0.001, F = 84.14, d.f. = 3, see Fig. 2), but also the stimulation current impacted the direction (p < 0.001, F = 16.39, d.f. = 1) as well as the interaction of current and electrode (p < 0.001, F = 6.94, d.f. = 3), indicating that the rotational change induced by a higher current was not consistent across electrodes.

The amplitude of evoked saccades did also significantly depend on the stimulated electrode and the stimulation current used, but not on the interaction of electrode and current, indicating that a higher stimulation current systematically increased saccade amplitudes (2-way ANOVA: p < 0.001, F = 17.03, d.f. = 3; p = 0.009, F = 7.48, d.f. = 1; p = 0.1, F = 2.17, d.f. = 3, for electrode, current and interaction, respectively).

Since the electrical stimulation was delivered concurrently with a behaviorally relevant task (i.e. the center-out saccade task), we next tested whether the parameters of saccades directly evoked by the stimulation changed as a function of task-instructed direction. Here, we pooled data from both current levels and then sorted by the direction of saccade target jump. We found that the frequency of evoked saccades did not depend on the direction of the instructed saccade (p = 0.323, odds ratio = 0.539; Fishers exact test). Also, while the effect of electrode location remained a significant factor to explain saccade direction, neither the direction of the instructed saccade, nor the interaction of electrode location and instructed direction explained the direction of the evoked saccades (2-way ANOVA for circular data: p«0.001, F = 51.13, d.f. = 3; p = 0.256, F = 1.32, d.f. = 1; p = 0.59, F = 0.64, d.f. = 3; for electrode location, instructed direction and interaction, respectively). Lastly, we also found no relationship between saccade amplitude and instructed direction, while the effect of stimulated electrode remained significant (2-way ANOVA: p < 0.001, F = 14.15, d.f. = 3; p = 0.31, F = 1.06, d.f. = 1; p = 0.68, F = 0.51, d.f. = 3; for electrode, instructed direction and interaction, respectively).

### Monkeys responded faster to contraversive jumps

To assess whether the monkeys responses to both saccade targets were comparable, we next analyzed only trials in which no electrical stimulation was delivered and the animals correctly made a saccade to the target. We calculated for each such control trial the saccadic onset latency as the time-point of the first saccade exceeding a velocity of 100 deg/s after the fixation point jump. We then tested for an effect of saccade direction (ipsi- vs. contralateral to array location). For this purpose, we normalized saccadic reaction times to speed by calculating the reciprocal of individual onset latencies^51^ and then tested with two sample T-tests for differences between contra- and ipsilateral saccades. For trials in which no electrical stimulation was delivered, we found a difference in saccadic reaction times between contra- and ipsiversive saccades (relative to array location), with contraversive saccades leading by a mean of 26 ms (median: 35 ms). This difference was highly significant across all trials and both animals (p ≪ 0.001, 2-sample T-test) and also within each individual animal (mean differences contra-ipsi of 17 ms and 35 ms for monkey E and S, respectively; both animals p ≪ 0.001). The difference was also present and significant both across and within each of the two animals when trials were grouped according to the stimulation current delivered on interleaved stimulation trials. A third, unimplanted animal measured in the same experimental setup did not show this bias (see Fig. S1). Using non-transformed saccadic onset latencies and/or performing non-parametric statistical tests (Wilcoxon rank-sum) did not qualitatively change our results.

### Stimulation lowered and prolonged saccadic latencies of voluntary saccades

To examine the behavioral effects of electrical stimulation of prefrontal region 8Av/45, we tested whether electrical stimulation during a visually guided saccade task changed saccadic onset latencies. Because both monkeys were significantly faster in initiating saccades towards the right hemifield (contralateral to stimulation), we analyzed the effects of electrical stimulation on contra- and ipsiversive saccades separately. Further, we only analyzed trials with saccade onsets between 100–400 ms relative to fixation-point jump and with saccade durations <75 ms, which well separated task-related from stimulation-induced saccades (see Fig. 1b). We also excluded all saccades recorded while stimulation was delivered to the seven electrodes found to evoke saccades during 250 μA stimulation in one animal (see Fig. 2b).

**Figure 3:**
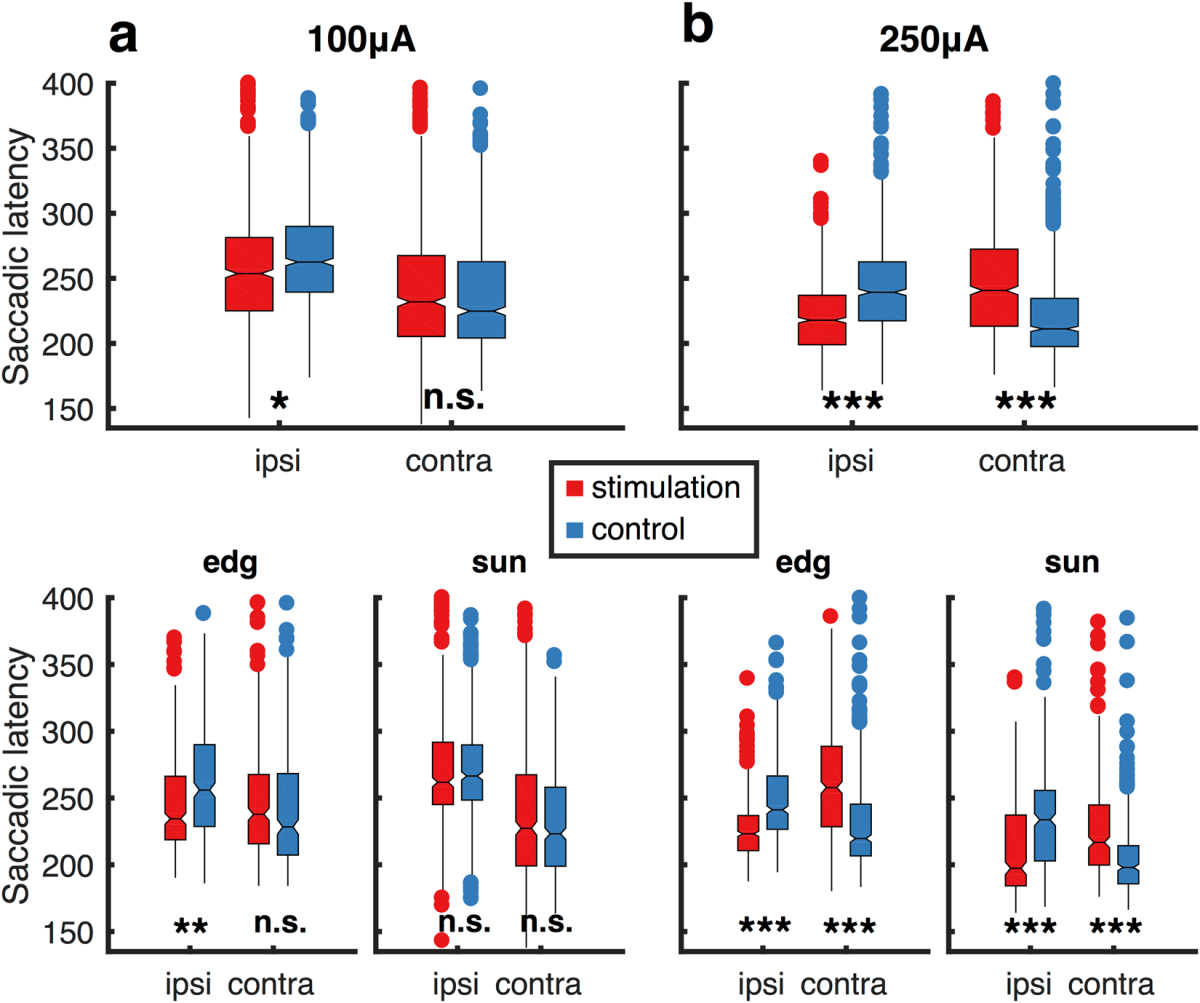
Latency effects. Electrical stimulation shortened saccadic reaction times for ipsiversive saccades and prolonged reaction times for contraversive saccades relative to randomly interleaved control trials without stimulation. Box and whisker plots of saccadic latencies for ipsi- and contraversive saccades from trials pooled for both animals **(top)** and for each individual animal **(bottom)**. During stimulation with 250 μA **(b**), stimulation significantly shortened ipsiversive saccade latencies and prolonged contraversive latencies. During stimulation with a current of 100 μA **(a)** only the effect on ipsiversive saccades was significant. Stars represent p-values of two-sample t-tests between groups, with * for p < 0.001, ** for p < 1e-6 and *** for p < 1e-12.

Across all remaining trials from both animals, we found that contraversive saccades were systematically delayed by about 25 ms (median of 30 ms) (p ≪ 0.001, 2-sample T-test as compared to trials without stimulation) for a stimulation current of 250 μA (see Fig. 3b). This effect was also significant within each monkey (both p ≪ 0.001, Fig. 3b lower panels). For stimulations with the low current of 100 μA, the average delay was reduced to about 4 ms (median: 7 ms) and was not significant (see Fig. 3a, n.s, p = 0.103, 2-sample T-test).

Ipsiversive saccades, on the other hand, were initiated faster in trials with electrical stimulation as compared to non-stimulated trials. In trials with a stimulation current of 250 μA, saccade latencies were on average 22 ms shorter than in trials without stimulation (median of −21 ms), and this difference was significant across and within both monkeys (all p ≪ 0.001, 2-sample T-tests, see Fig. 3b). For the lower current this effect was still present and significant, but smaller (mean of −9 and median of −10 ms; p < 0.001, 2-sample T-test, see Fig. 3a; p = 0.026 for monkey S and p < 0.001 for monkey E).

Again, these results were robust across statistical tests, as using non-transformed saccadic onset latencies and/or performing non-parametric statistical tests (Wilcoxon rank-sum) did not qualitatively change our results.

### Systematic spatial organization of latency effects

We next tested, whether the effects of stimulation on saccadic latencies varied significantly with stimulation site. For this purpose, we limited our analysis to trials with the stronger stimulation current (250 μA) and their interleaved control trials and divided the electrode arrays (covering a cortical surface of 2 mm^2^) into equally sized caudal and rostral electrode groups. Caudal electrodes were located closer to the concavity of the arcuate sulcus (i.e. towards FEF), while rostral stimulation sites were located further anterior, along the principal sulcus (i.e. towards VPA).

**Figure 4:**
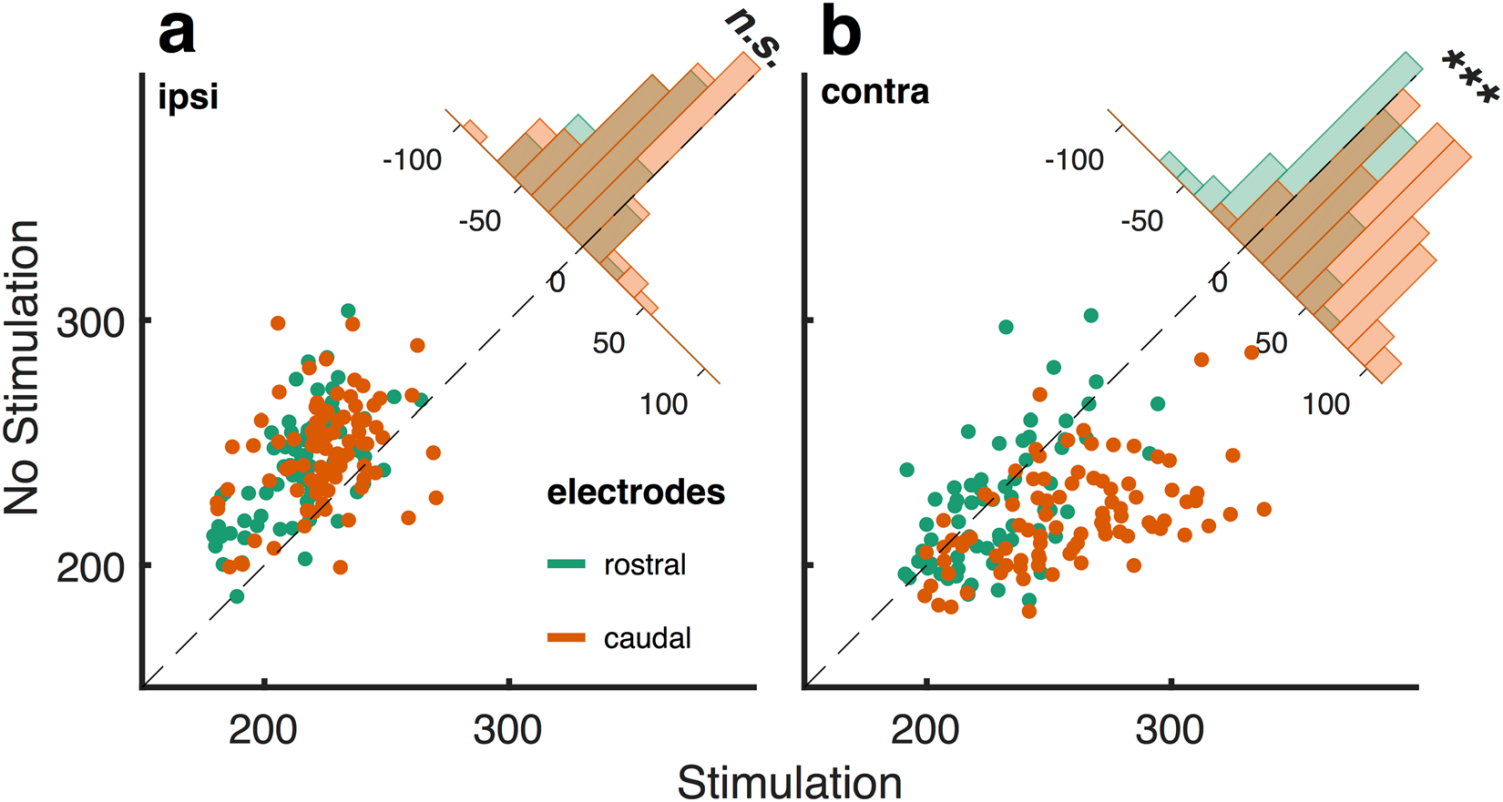
Latency effects depend on electrode location. Contraversive saccades were initiated later in stimulation trials compared to control trials, but only when stimulation was delivered to caudal electrodes closest to the Frontal Eye Fields. For each stimulated electrode we plotted the averaged saccadic latencies for 250 μA stimulation trials against control trials. Electrodes were divided into rostral and caudal groups for ipsiversive **(a)** and contraversive **(b)** saccades. Corner histograms represent the latency effects for each group. Stars indicate significant differences with p < 1e-12.

For ipsiversive saccades and across animals, this division of stimulation sites did not reveal differences in the effect of stimulation on saccadic latencies: both the stimulation of rostral and caudal electrodes caused a significant reduction of saccadic latencies, compared to non-stimulated trials (both p ≪ 0.001, paired-sample T-tests). We also did not detect differences in the size of the effect between rostral and caudal electrode groups (p = 0.147, 2-sample T-test, see Fig. 4a).

Contraversive saccades, on the other hand, were differently affected by electrode location, with caudal stimulation prolonging saccadic latencies, compared to non-stimulated trials, while this effect was not significant for rostral stimulation sites (p ≪ 0.001 and p = 0.038, for caudal and rostral electrode groups, respectively; Bonferroni adjusted alpha = 0.0125, paired-sample T-tests). In line with this observation the effects of stimulation on saccadic latencies were significantly different for caudal and rostral electrode groups (p ≪ 0.001, 2-sample T-test, see Fig. 4b).

### Stimulation did not influence saccade speed or amplitude, but tended to decrease accuracy of contraversive saccades

For each saccade that was included in the previous analysis (see Fig. 3), we also calculated the peak velocity and then analyzed whether stimulation had an effect on saccade speed. Mean velocity differences between stimulated and non-stimulated trials were minimal across monkeys (i.e. across stimulation currents 1.98 deg/s for contraversive and 6.28 deg/s for ipsiversive saccades, p = 0.54 and p = 0.12 respectively, 2-sample T-tests). Nonetheless, we analyzed all combinations of both current levels and both saccade directions within and across both monkeys. After correction for multiple comparisons (n = 8, Bonferroni corrected alpha = 0.0063), we found that none of the comparisons reached statistical significance (see also Fig. S2).

Changes in saccade amplitudes were only significant in animal S, and only for contraversive saccades during high current stimulation. Here, stimulation marginally shortened saccades (mean of −0.13 degrees; p = 0.002, 2-sample T-test), otherwise we observed no significant effects of stimulation on saccade amplitudes and no consistent trends across animals (see also Fig. S3).

For the euclidian distance between saccade endpoints and saccade target, on the other hand, we observed an effect of stimulation-induced deterioration of end-point accuracy. On average and across animals and currents, saccadic endpoints were 0.04 visual degrees further from targets on stimulated trials as compared to non-stimulated trials (p = 0.003, 2-sample T-test). This accuracy difference also tended to be larger when stimulation currents were higher (differences of 0.03 deg and 0.06 deg; p = 0.168 and p = 0.003 respectively for 100 μA and 250 μA stimulation, 2-sample T-tests). However, those relatively weak effects originated only from the data of one monkey and were effectively absent in the other (see also Fig. S4)

## Discussion

In this study we report the immediate and behavioral effects of unilateral electrical stimulation of macaque lateral prefrontal region 8 Av/45 using chronically implanted 96-channel microelectrode arrays. Monkeys were engaged in a goal-directed center-out saccade task and stimulation was delivered on 50% of the trials, sequentially to each single electrode and concurrently with the fixation-point jump.

Although we found that stimulation, especially using higher currents, sometimes *directly* evoked saccades in one monkey, the most prominent stimulation effects constituted *behavioral* changes related to the task the animals were carrying out. Here, we showed that consistently across both animals, saccades that were directed to either hemifield were affected: Electrical stimulation prolonged saccadic latencies for contraversive, and shortened saccadic latencies for ipsiversive saccades, compared to saccades in the same direction during non-stimulated trials. Effects on contraversive saccades were most pronounced for caudal stimulation sites close to the Frontal Eye Fields (FEF) and to those sites that directly evoked saccades (in one animal). Rostral stimulation close to the ventral prearcuate region (VPA^42^) did not impact contraversive saccades, but shortened reaction times for ipsiversive saccades, similar to caudal stimulation. This pattern of results is most compatible with a stimulation-induced biasing of stimulus processing that accelerates the detection of saccade targets presented ipsilateral to stimulation through a disruption of contralaterally deployed top-down attention.

For a spatially confined patch of electrodes in one animal, electrical stimulation of 8 Av/45 directly evoked saccades. Saccadic endpoints were exclusively located in the visual field contralateral to the stimulated hemisphere. Higher currents evoked saccades in slightly different directions, but the predominant effect of current was an increase in saccadic amplitude. Our data for this spatially confined cortical patch close to FEF thus support a model of amplitude- and direction-based saccade encoding - unlike the fixed-endpoint encoding expected for adjacent FEF^18,52^.

Further, even though our stimulation protocol closely resembled that of recent microstimulation studies of FEF^23,24^, we robustly elicited saccades only when using currents about an order of magnitude larger than typical saccadic thresholds for FEF^18,22^. We thus argue that our stimulation electrodes were indeed located at the junction between areas 8Av and 45, and not in adjacent (functionally defined) FEF. This is consistent with a previous report by Schall *et al*.^53^, demonstrating that electrical microstimulation of the peri-arcuate region can evoke saccades, but typically only for stimulation sites very close to the FEF. Indeed, 8Av was not previously described as a node in the saccade generation network, although according to a recent study, the direction of upcoming saccades can be decoded from 8Av/45 single neuron activity. In their report, Boulay *et al*.^54^ described single units that encoded a saccade direction just after saccade target onset, but sometimes changed their selectivity during a delay period. This points to an involvement of 8Av/45 in both saccade target representation and its sensorimotor transformation, rather than the encoding of the actual motor plan – an interpretation that is also supported by our stimulation data.

Our monkeys performed a simple center-out saccade task with a deterministic timeline for each single trial. Only two options existed for the appropriate motor response – a left- or rightward saccade. Because of this simplicity, it is very likely that the monkeys were planning both possible movements well before one of them was selected for execution by the fixation point jump^55,56^. This is in line with the preference of both monkeys for contraversive (rightwards) saccades in control trials without stimulation, since this effect may originate from the experience of the two animals, which were both extensively trained on an attentional task with stimuli exclusively presented in the right hemifield. In fact, we collected control data from a third animal that did not receive such previous training and this animal, although measured in the same experimental setup, did not show a bias for a specific saccade direction. However, it was also not implanted with a prefrontal array. Thus, we cannot rule out a (constant) effect of the implantation on saccadic reaction times. As a result, for stimulation trials, we only analyzed changes in reaction times relative to not stimulated control trials with the same saccade target.

We electrically stimulated concurrently with the fixation point jump and found that this stimulation both increased contraversive and decreased ipsiversive saccadic reaction times (RT). We observed no statistically significant effects on saccade speed, amplitude or accuracy, which renders a stimulation-induced motor impairment for specific saccades unlikely.

Instead, our results are in line with previous research showing that stimulation of non-saccade sites of FEF prolongs saccadic RTs^57,58^, consistent with an impairment of unilateral cue processing. However, contrary to those previous observations, we show that stimulation of 8Av/45 – unlike stimulation of FEF – also significantly shortens RTs for ipsiversive saccades. Such a pattern of results can best be accounted for by a stimulation-induced influence on motor plan selection, among the two possible options available to the monkeys.

We therefore argue that stimulation interferes with the execution of saccade plans, either by altering the attentional weighting of targets presented in the two hemifields through changes of the attentional state of the animals, or by directly influencing the decision process, selecting a specific saccade plan for execution. In both cases, stimulation likely caused an imbalance (e.g. attentional bias) between the two hemispheres, disrupting the execution of contraversive and thereby favoring the execution of ipsiversive saccades.

The above conclusion is further supported by the fact that contraversive, but not ipsiversive saccade latency changes depended on the stimulation location. Only when the caudal portion of 8Av/45 (sometimes called ‘preFEF’^58^) was stimulated, contraversive saccade execution was delayed relative to control trials. For ipsiversive saccades, on the other hand, a decrease in saccadic latency was caused by stimulation regardless of the position of the stimulation electrode within 8 Av/45.

Such a pattern can be explained by a stimulation-induced disruption of a strongly lateralized system – such as the cortical networks responsible for the deployment of spatial attention^59,60^. Our data then suggest that caudal stimulation of 8Av/45 induces more salient^61^ alternatives to the contralateral saccade target instructed by the task at hand, because of the more direct proximity to spatiotopically organized FEF. Rostral stimulation, close to (non-spatial) VPA, also influences the deployment of attention to contraversive target locations, but in a less spatially precise manner and thus causes weaker effects on saccadic latencies. For ipsiversive latencies this difference is less relevant, because here the factor determining saccadic latency is whether the deployment of attention for the (competing) contralateral hemifield is disrupted – not how well this artificially induced saliency competes with the contralateral focus of spatial attention.

However, there is an important alternative explanation for the observed effects. Instead of interfering with the saliency of possible saccade targets, stimulation of area 8Av/45 might have an impact on the perceived behavioral *value* of contralateral saccades. Here, instead of lowering the (visual) saliency of contralateral saccade targets, lowering the perceived value of saccades towards this hemifield (e.g. by lowering the expected reward) would directly impact decision thresholds and thus cause the observed re-balancing of saccadic latencies^62,63^.

In fact, information about the value of specific actions are also reflected in neuronal activity measured from primate lateral prefrontal cortex^64–66^, although the primate reward/value network is thought to involve mainly medial and orbitofrontal areas^67,68^. It is also not clear why stimulation close to the FEF should cause a greater reduction of the values of contralateral saccades while the value of ipsilateral saccades remains unchanged by stimulation location.

Considering the previously hypothesized role of the stimulated area in the generation of top-down attentional signals, and the likely involvement of adjacent and interconnected FEF in the deployment of spatial attention to visual cortex, our data provide better evidence for a model in which electrical stimulation directly affected the deployment of visual attention and – therewith – the orchestration of activity in visual cortex.

In summary, we have shown that electrical stimulation of 8Av/45 robustly interferes with a concurrent goal-directed task, here a center-out, left-right saccade task. The observed behavioral effects were complex and differed from effects observed during stimulation of adjacent FEF. Our data support the theory that the neural activity of 8Av/45 is related to a general guidance of attention and task-rule encoding. Thus, our results support a role of 8 Av/45 as a node of cortical networks subserving the deployment of attention and complex rule-based decision-making.

## Methods

Research with non-human primates represents a small but indispensable component of neuroscience research. The scientists in this study are aware of and committed to the great responsibility they have in ensuring the best possible science with the least possible harm to the animals^69^.

### Subjects

Two adult male macaque monkeys (macaca mulatta, both 13 years old, weights 13 kg and 8 kg) were implanted with custom-made titanium headposts and 4 mm^2^ × 1.5 mm 96-channel microelectrode arrays (Blockrock, USA). Arrays were placed on the surface of each animal’s left lateral prefrontal cortex, just ventral to the caudal end of the principal sulcus, identified based on structural MRI scans and anatomical visual landmarks (during implantation). The arrays were then pneumatically inserted into the cortex and permanently remained there with the dura and skull closed above them. Surgeries were performed aseptically under gas anesthesia using standard techniques, including appropriate peri-surgical analgesia and monitoring.

The animals were pair-housed in facilities of the German Primate Center (DPZ) in accordance with all applicable German and European regulations. The facility provides the animals with an enriched environment (incl. a multitude of toys and wooden structures^70^), natural as well as artificial light and access to outdoor space, exceeding the size requirements of European regulations. During the study the animals had unrestricted access to food and fluid, except on the days where data were collected or the animals were trained on the behavioral paradigm. On these days, animals had access to fluid through their performance in the behavioral paradigm. Some of the behavioral training was conducted in the animal facility, using a kiosk-type system^71^. Throughout the study the DPZ’s veterinarians, the animal facility staff and the lab’s scientists monitored the animals’ psychological and veterinary welfare. The two animals were healthy at the conclusion of our study and were subsequently (and previously) used in other studies.

All experiments of this study were performed in accordance with relevant guidelines and regulations and approved of by the responsible regional government office (Niedersaechsisches Landesamt fuer Verbraucherschutz und Lebensmittelsicherheit (LAVES)) under the permit number 3392 42502-04-13/1100.

### Apparatus

Monkeys were seated head-fixed in a primate chair at a viewing distance of 102 cm from a back-projection screen (dnp Black Bead, Denmark, 171.5 × 107.2 cm image size). Two projectors (Projection Design F22, Norway) and two sets of linear polarizing filters were used to display stereoscopic stimuli with a 60 Hz refresh rate and a resolution of 1920 × 1200 pixels. Stimuli were presented perimetrically on a virtual spherical bowl at zero disparity.

Eye position was recorded binocularly with an Eyelink 1000 system (SR-Research, Canada) at a sample rate of 500 Hz. The eye position system was calibrated with a custom 3D calibration routine prior to each experimental session. The experiment was controlled by an Apple computer (Mac Pro 2010) running the open-source software MWorks 0.5 (mworks-project.org).

### Stimuli and Procedure

A red fixation point (0.5 degrees diameter) central to a random dot stereogram (RDS; 5 × 5 degrees, displayed at 0° disparity) instructed the monkeys to maintain their gaze within a sphere with a radius of 2 degrees around the fixation point. Monkeys were required to foveate this point and maintain their gaze for 800 ms, after which the fixation point and RDS jumped horizontally to a location 7 degrees lateral to their previous location, with left and right jumps pseudo-randomly interleaved. Monkeys were required to respond to the jump by making a saccade to the new location of the point. Once the fixation point was foveated again, they received a liquid reward and the trial was terminated (see Fig. 1a).

For each electrode of the prefrontal arrays and each stimulation current, we ran a block of 20 trials, with electrical stimulation delivered on 50% of the trials.

### Electrical stimulation

In stimulation trials, we delivered 10 biphasic pulses with a frequency of 200 Hz (see Fig. 1d) to a single electrode of the monkeys’ 96-channel electrode array. The waveform was produced by a Grass S88X stimulator and amplified by a WPI A365 stimulus isolator. We measured the voltage drop over a 1kΩ shunt resistor in series with the stimulated electrode using a digital oscilloscope and thereby estimated and adjusted the electrical current delivered to the electrode to either 100 or 250 μA (read as half peak-to-peak values). Using this method, we were also able to estimate the impedances of each single electrode, compare them to their nominal values (typically 200–400 kΩ) and exclude any electrodes from the analysis that seemed disconnected or broken (see also Fig. 2 small inserts).

Electrical stimulation was initiated always concurrently with fixation point jump and delivered pseudo-randomly on 50% of all trials.

### Data Analysis and Availability

The data analysis was performed with MATLAB R2016b using custom scripts. For the analysis of circular data, we used the CircStat2012a toolbox^50^ and Figs 1b, 3 and 4 were plotted using the gramm toolbox (version 258a4fa)^72^.

The datasets generated and analyzed during the current study are available from the corresponding author on reasonable request.

## Electronic supplementary material

Supplementary Information
