# Supplementary material for: Electrical stimulation of macaque lateral prefrontal cortex modulates oculomotor behavior indicative of a disruption of top-down attention
Source: Sci Rep. 2017 Dec 18;7:17715. doi: 10.1038/s41598-017-18153-9 (PMC5735183; doi:10.1038/s41598-017-18153-9)

Supplementary information for:

Electrical stimulation of macaque lateral prefrontal cortex modulates oculomotor behavior indicative of a disruption of top-down attention

by Philipp Schwedhelm, Daniel Baldauf and Stefan Treue

Figure S1

Saccadic onset latencies of a control animal do not show an effect of saccade direction.

We collected a single session of the center-out, left-right saccade task from a third animal, that had no prior exposure to behavioral tasks requiring attention to be directed to the contralateral hemifield (relative to the electrode arrays of the two animals used for the main experiment).
For this animal, which was recorded in the same experimental setup, onset latencies for left- and rightward saccades were not different ($p=0.176$, 2-sample T-Test).
Data illustrated by box and whisker plots with corresponding raw data points in the background. The shaded 'violin'-area represents the probability density estimates (based on a normal kernel) for the raw saccade onset data (in ms).

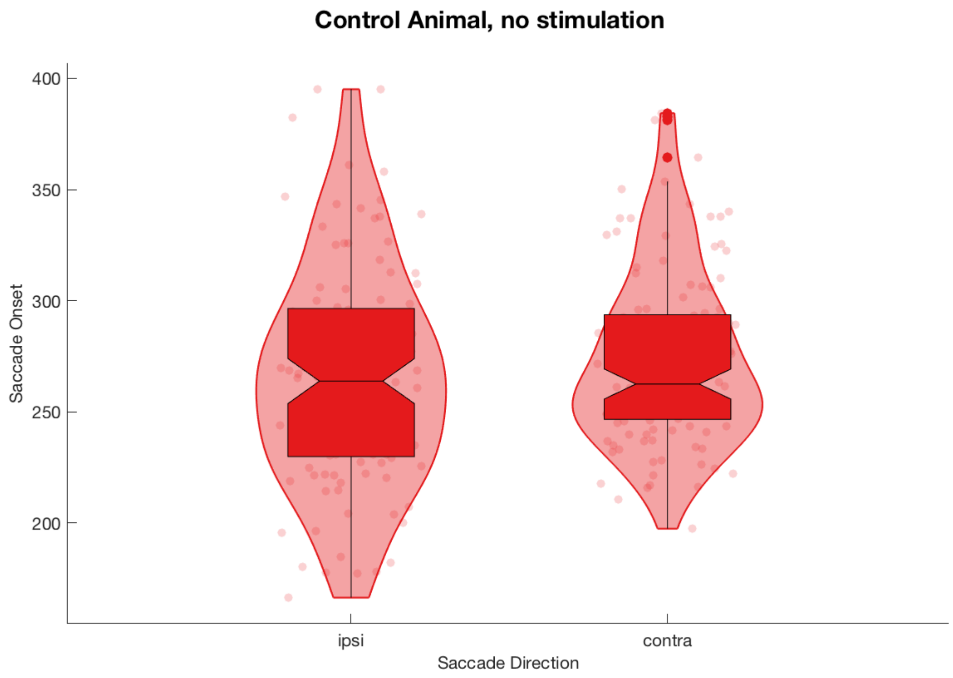

Figure S2

Peak velocities do not change with stimulation

We compared peak velocities of saccades with and without electrical stimulation for two currents (in two columns). None of the comparisons with data pooled across animals (top row) and within each individual animal (bottom row) reached statistical significance.

Data is illustrated by box and whisker plots with corresponding raw data points in the background. The shaded 'violin'-area represents the probability density estimates (based on a normal kernel) for the raw peak velocity data (in deg/s).

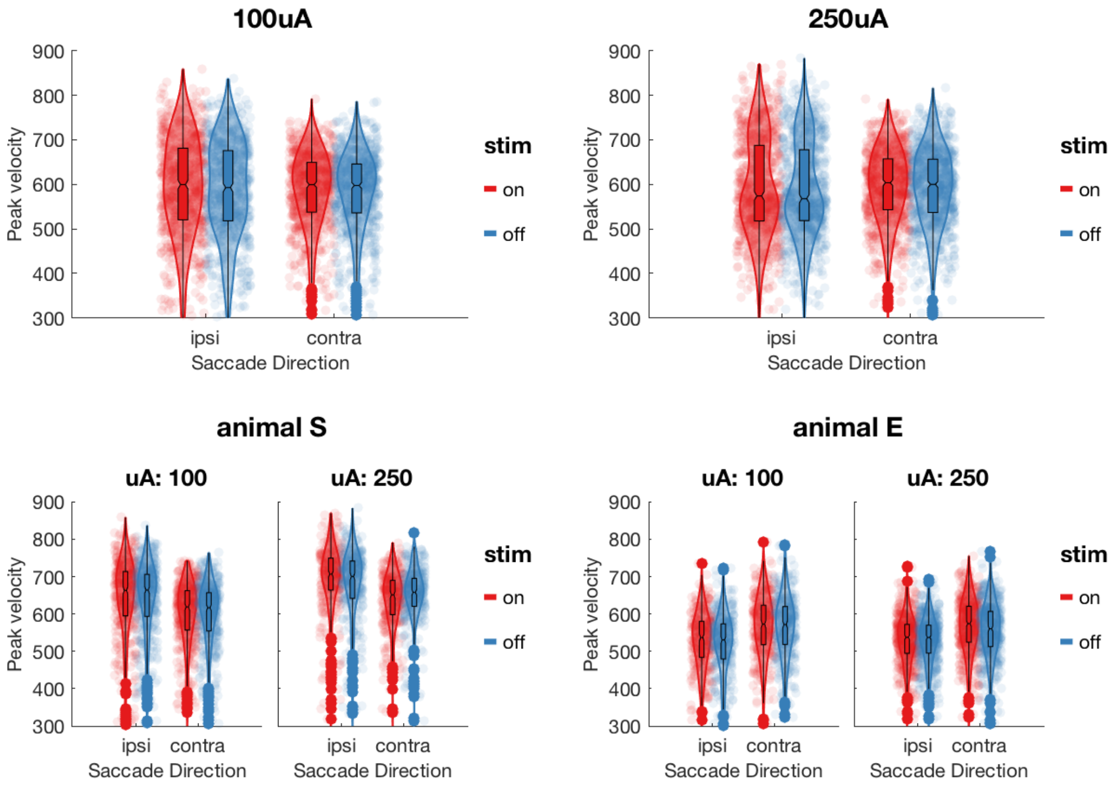

Figure S3

Stimulation marginally shortens saccadic amplitudes

We compared the amplitudes of saccades with and without electrical stimulation for two currents (in two columns). None of the comparisons with data pooled across animals (top row) reached significance. However, for animal S (bottom left), contraversive saccades were marginally shorter during high current stimulation (p=0.002, see also main manuscript). This effect was not present for animal E (bottom right).
Data is illustrated by box and whisker plots with corresponding raw data points in the background. The shaded 'violin'-area represents the probability density estimates (based on a normal kernel) for the raw amplitude data (in visual degrees).

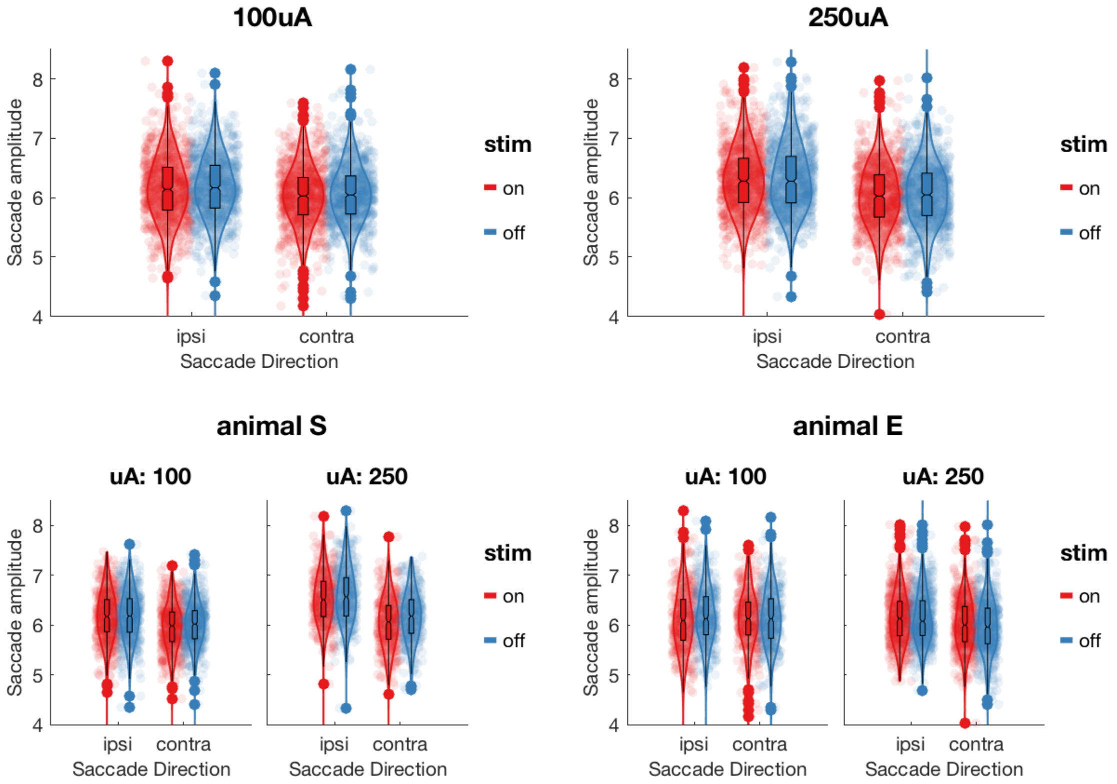

Figure S4

Stimulation impacts saccadic accuracy in one animal

We compared the landing error of saccades with and without electrical stimulation for two currents (in two columns). Across animals (top row), the Euclidian distance between the saccade end-points and the saccade target was slightly (but significantly) bigger for contraversive saccades during high current stimulation (top right plot). This effect originated from animal S (bottom left plots) and was not present in animal E (lower right plots).
Data is illustrated by box and whisker plots with corresponding raw data points in the background. The shaded 'violin'-area represent the probability density estimates (based on a normal kernel) for the raw Euclidian distances between saccade target and saccade end-point (in visual degrees).

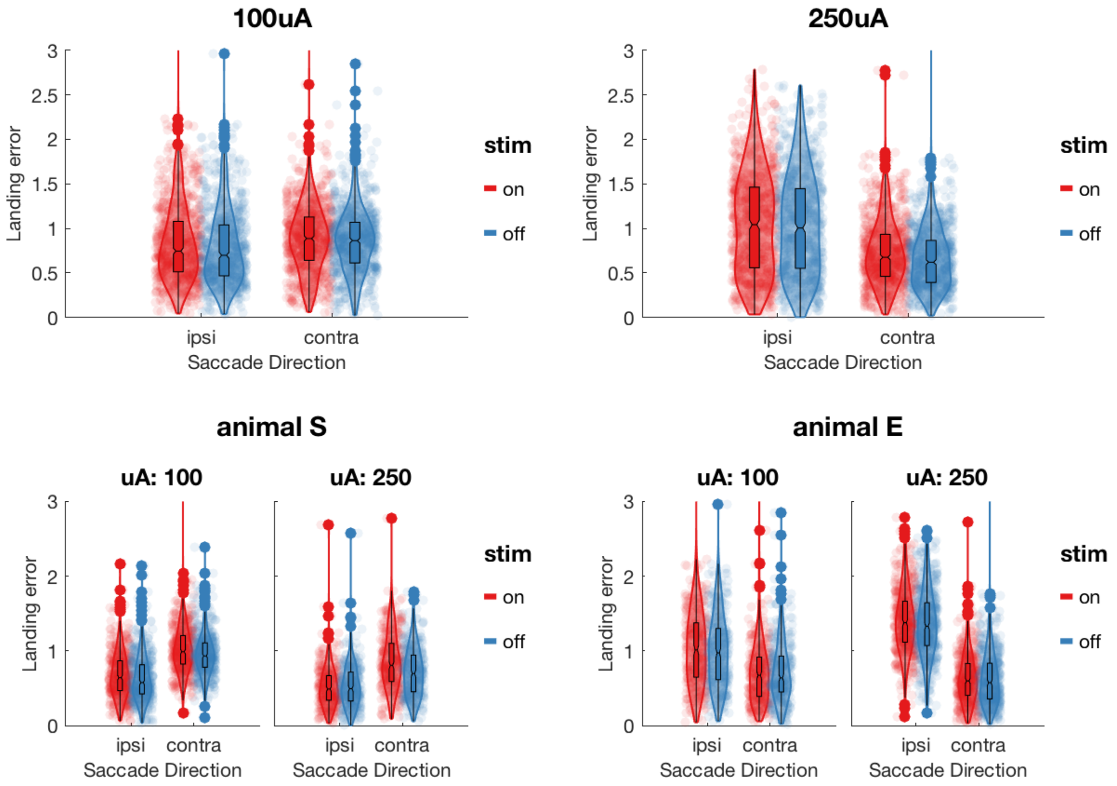

Supplement: Supplementary file 1 — Supplementary Information [file 41598_2017_18153_MOESM1_ESM.pdf]
